# Endometrial Cytology as a Method to Improve the Accuracy of Diagnosis of Endometrial Cancer: Case Report and Meta-Analysis

**DOI:** 10.3389/fonc.2019.00256

**Published:** 2019-04-24

**Authors:** Qing Wang, Qi Wang, Lanbo Zhao, Lu Han, Chao Sun, Sijia Ma, Huilian Hou, Qing Song, Qiling Li

**Affiliations:** ^1^Center for Single-Cell Biology, First Affiliated Hospital, Xi'an Jiaotong University, Xi'an, China; ^2^Department of Obstetrics and Gynecology, First Affiliated Hospital, Xi'an Jiaotong University, Xi'an, China; ^3^Guipei 77, Health Science Center, Xi'an Jiaotong University, Xi'an, China; ^4^Department of Pathology, First Affiliated Hospital, Xi'an Jiaotong University, Xi'an, China

**Keywords:** cytology, histology, endometrial cancer, diagnosis, atypical hyperplasia

## Abstract

More and more researchers have reported that dilatation and curettage (D&C) or Pipelle had low accuracy, high misdiagnosis, and insufficient rate. Endometrial cytology is often compared with histology and seems to be an efficient method for the diagnosis of endometrial disorders, especially endometrial cancer. We report a case of misdiagnosed endometrial cancer by D&C, but with a positive cytopathological finding. Following that, a meta-analysis including 4,179 patients of endometrial diseases with cyto-histopathological results was performed to assess the value of the endometrial cytological method in endometrial cancer diagnosis. The pooled sensitivity and specificity of the cytological method in detecting endometrial atypical hyperplasia or cancer was 0.91[95% confidence interval (CI) 0.74–0.97] and 0.96 (95% CI 0.90–0.99), respectively. The pooled positive likelihood ratio and negative likelihood ratio was 25.4 (95% CI 8.1–80.1) and 0.10 (95% CI 0.00–0.30), respectively. The diagnostic odds ratio which was usually used to evaluate the diagnostic test performance reached 260 (95% CI 36–1905). So we recommend that D&C and Pipelle are still practical procedures to evaluate the endometrium, cytological examinations should be utilized as an additional endometrial assessment method.

## Introduction

Endometrial cancer is becoming the primary reason of female deaths of genital track cancer in developed countries (1). Dilatation and curettage (D&C), as the traditional gold standard procedure for diagnosing endometrial cancer, is painful, expensive, requires general anesthesia and has a high rate of misdiagnosis (2). It has been reported that less than half of the uterine cavity is curetted in 60% of cases (3), and over 40% of women with complex atypical hyperplasia as a preoperative diagnosis have a final confirmation of endometrial cancer during hysterectomy (4, 5). Endometrial cytology is recently reported as a useful diagnostic method with high sensitivity and specificity in detecting endometrial malignancies (6–9), but no meta-analysis, which is considered more credible, has yet been performed to evaluate the diagnostic accuracy of endometrial cytology for endometrial carcinoma compared with histological diagnosis.

Here, we report a case of misdiagnosed endometrial cancer by D&C, but with a positive cytopathological finding. The patient has provided her written informed consent for the publication of this manuscript and any identifying images or data. After searching on PubMed, we believe it is the first case report of a misdiagnosis of endometrial cancer detected by cytopathology. Following this, a random-effects meta-analysis including 4,179 patients with both cytopathological and histopathological results was performed to assess the value of the endometrial cytology method in the diagnosis of endometrial atypical hyperplasia or cancer.

## Case Report

**Figure 1:**
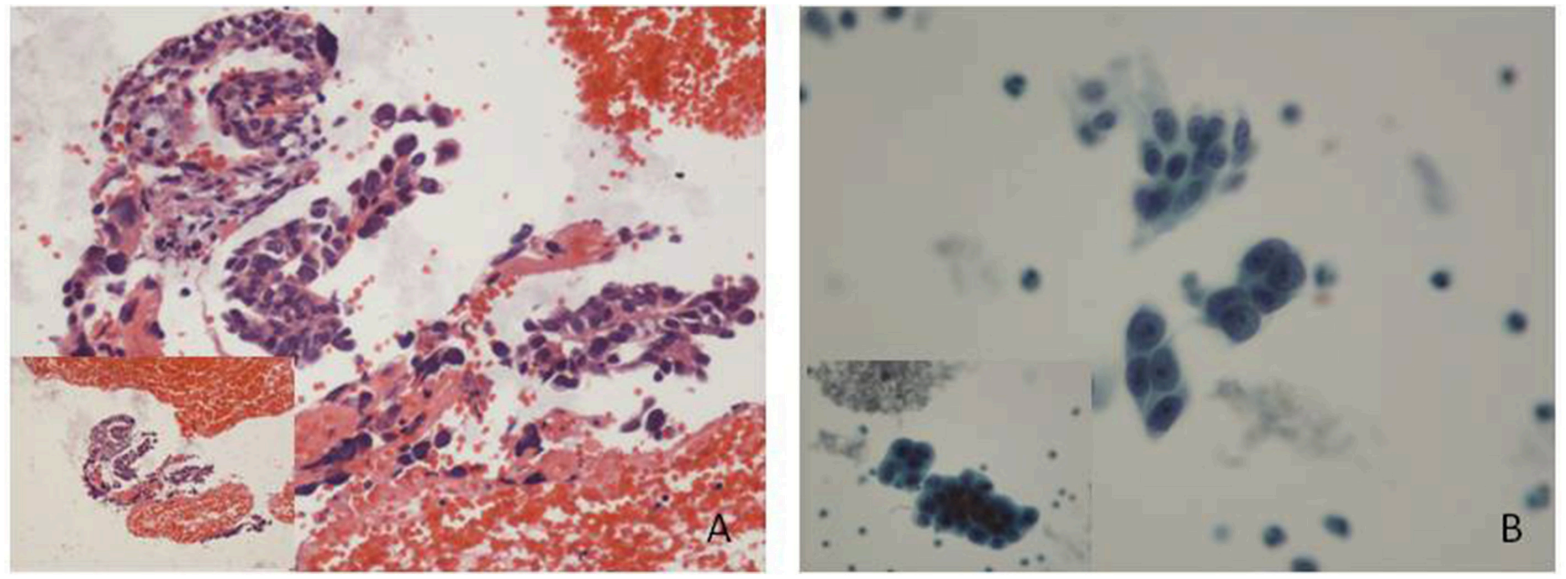
Histological and cytological images. **(A)** Some papillary arranged epithelial dysplasia cells could be found in plenty of blood cells, with no tissue structure. (Hematoxylin-eosin staining; original magnification x10). **(B)** Endometrial carcinoma cells: cell clumps with irregular protrusions were rich in dimensional sense. Variable sizes, different shapes and hyperchromatic nuclei showed a loss of polarity within the epithelial sheet with irregularly clumped chromatin (Papanicolaou stain; original magnification x 20).

A 60-year-old post-menopause female, from Baoji City of the Shaanxi province in China, went to a local hospital complaining of abnormal uterine bleeding for 2 months. No high risk factor for endometrial cancer was observed, such as genetic factors, obesity, diabetes, a history of tamoxifen use and so on. Curettage was performed with a histopathological diagnosis of complex hyperplasia endometrium. No medicine or therapeutic curettage was effective for her with a continued bleeding. Her type B ultrasound in Shaanxi Provincial People's hospital showed a 0.8 cm-thick endometrium. Then, she turned to the First Affiliated Hospital of Xi'an Jiaotong University for further treatment. After written informed consent, she volunteered to get cytological endometrial samplings by Li Brush (Xi'an Meijiajia Bio-Technologies Co. Ltd., China, 20152660054) for cytological examination before D&C. Her histopathological report revealed that papillary epithelial hyperplasia was found, and cancer was a concern according to the structure of tissue but could not be diagnosis due to insufficient tissue (Figure 1A). Meanwhile, the cytopathological report revealed that some malignant cells were found (Figure 1B). Her serum markers showed high serum carbohydrate antigen 19-9 (CA19-9, 42.08 U/ml) and squamous cell carcinoma antigen (SCC, 6.10 ng/ml). A diagnostic laparoscopic hystero-salpingo-oophorectomy was performed and the patient was converted to a laparotomy when intraoperative frozen section examination revealed an endometrial serous carcinoma with ovarian metastasis. Omentum resection, pelvic lymphadenectomy and para-aortic nodes dissection were performed. She was finally diagnosed with stage IIIc endometrial serous carcinoma.

## Materials and Methods

### Source of Material

We searched the PubMed and Embase databases with the heading terms and keywords as “cytology” and “endometrial” from Jan 1, 1995 to June 1, 2018. Then, the results were manually selected for studies to include and repeatedly checked by a second investigator. We searched the full-text articles about the comparison of cytological results and the histological results in endometrial samples.

### Standard of Inclusion and Exclusion

All candidate studies were evaluated and extracted by two independent investigators. Inclusion criteria: (1) patients were diagnosed by histopathological and cytological examination; (2) the histopathological results were paired with cytological results; (3) sufficient information was provided to conduct a statistical analysis; (4) endometrial cells were sampled by endometrial brushes; (5) studies were limited to human trials and published in English. Exclusion criteria include: (1) news, abstracts, case reports, letters, commentaries, and reviews studies; (2) other kinds of endometrial cells sampler like endometrial aspiration cytology; (3) different cytopathology report formats with others, that made it hard to re-group and analyze; and (4) studies with different positive result definition or duplicate data.

### Data Extraction

We set atypical hyperplasia and endometrial carcinoma as the positive results and the others as the negative results, including normal endometrium, non-atypical hyperplasia, endometrial polyp, simple endometrial hyperplasia, complex endometrial hyperplasia and so on.

Two investigators separately extracted the following information from each research: the name of the first author, year of publication, cytological sampling method, cytological specimen preparation, histological sampling method, number of patients enrolled, and true positive (TP), false negative (FN), false positive (FP), and true negative (TN) results. Any discrepancies between the two investigators were discussed by all the authors.

### Quality Assessment

A quality assessment of eligible studies was evaluated using the quality assessment of studies of diagnostic accuracy included in systematic reviews-2 (QUADAS-2). There were 13 questions (each of which was scored as yes, no, or unclear): (1) Was a consecutive or random sample of patients enrolled? (2) Was a case–control design avoided? (3) Did the study avoid inappropriate exclusions? (4) Were the cytopathological diagnoses interpreted without knowledge of the results of the gold standard (histopathological diagnosis)? (5) whether the blind method was used for pathologists? (6) Was the histopathological diagnosis likely to correctly classify the target condition? (7) Was there an appropriate interval between the cytological sampling and histological sampling? (8) Did all patients receive the histopathological diagnosis? (9) Were all patients included in the analysis? (10) Whether the diagnostic test steps were detailed? (11) Were there concerns that the included patients and setting do not match the review question? (12) Were there concerns that the target condition as defined by the gold standard does not match the question? (13) Were there concerns that the index test, its conduct, or its interpretation differ from the review question?

### Statistical Analysis

The publication bias was checked by a Deeks funnel plot, and *P* < 0.05 was considered a significant publication bias. Statistical heterogeneity was detected by a Q test and an inconsistency index (*I*^2^), with significant heterogeneity set at *P* ≤ 0.05 and *I*^2^ > 50%. If there was no significance in heterogeneity (*P* > 0.05), a fixed effects model was chosen. If it was the opposite (*P* < 0.05), a random effects model was chosen.

According to TP, FN, FP and TN results, we calculated sensitivity, specificity, positive predictive value (PPV), negative predictive value (NPV), positive likelihood ratio (PLR) (>10 suggested strong concordance), negative likelihood ratio (NLR) (<0.1 suggested strong concordance), diagnostic odds ratio (DOR). PLR was calculated as: positive likelihood ratio = sensitivity/(1–specificity). NLR was calculated as: negative likelihood ratio = (1–sensitivity)/specificity. DOR was estimated by the Mantel-Haenszel formula. All statistical analyses, including 95% Confidence Interval (CI) were performed using STATA software (version 12.1, StataCorp LP) with the Midas module.

## Results

### Search Results

**Figure 2:**
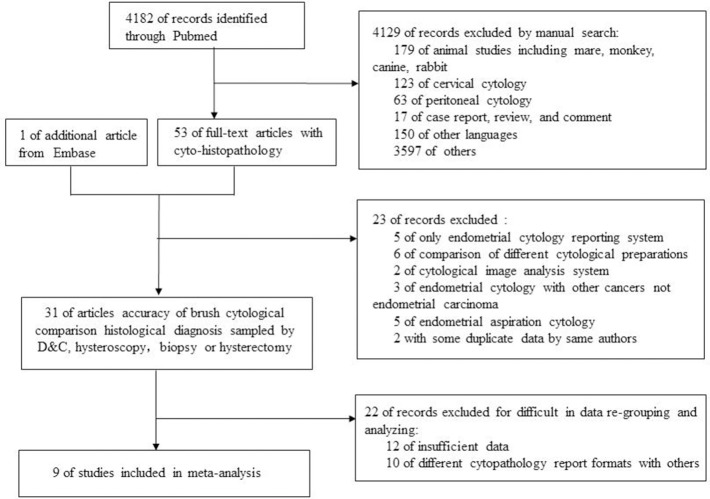
Study selection process.

After searching on PubMed and Embase, 9 of 4,182 studies were included in meta-analysis. Figure 2 showed a flow diagram of the selection process. All data in researches were screened rigorously by our team.

### Basic Characteristics of Studies

Our analysis included 9 eligible studies, which were shown in Table 1. In total, 2 studies were from Italy, 2 from the USA, 1 from China, 1 from Japan, 1 from England, 1 from Indonesia, and 1 from Greece. A total of 4,179 patients were included. Different endometrial brushes were used in these 9 studies, including the Tao brush (2), Endoflower (2), Endogyn (1), Cytobrush (1), and Uterobrush (1), and 1 study used six different devices. In all, 8 studies prepared the cytology specimens with a liquid-based cytology, and 1 study used the conventional way. In sum, 2 studies compared the cytological results to the D&C results, 3 studies compared the cytological results to the hysterectomy results, 2 studies compared the cytological results to the hysteroscopy and biopsy results, 1 study compared the cytological results to the biopsy or D&C results and 1 study compared the cytological results to the biopsy, D&C or hysterectomy results. Additionally, 5 studies research the pre/post-menopausal patients, 2 studies researched peri/post-menopausal patients, 1 study researched post-menopausal patients, and in 1 study, the menopause situation was unknown.

**Table 1:** Study characteristics of the nine included studies on the diagnostic accuracy of endometrial cytological sampling.

**Study**	**Year/country**	**Cytological sampling and preparation**	**Histologic sampling**	**Menopausal status**	**Sample size**	**TP**	**FP**	**TN**	**FN**	**PPV %**	**NPV %**
Maksem et al. (10)	1997 USA	Tao brush/ LBC	Hysterectomy	Pre/post	100	18	1	81	0	94.7	100.0
Garcia et al. (11)	2003 England	Uterobrush/ LBC	Biopsy/D&C/ hysterectomy	Pre/post	60	7	2	49	2	77.8	96.1
Papaefthimiou et al. (12)	2005 Greece	Endogyn/ LBC	Hysterectomy	Peri/post	491	191	5	292	3	97.4	99.0
Andrijono et al. (13)	2005 Indonesia	Cytobrush/ LBC	D&C	Peri/post	45	5	3	24	13	62.5	64.9
Buccoliero et al. (14)	2007 Italy	Endoflower/ LBC	Hysteroscopy and biopsy	Pre/post	531	29	0	501	1	100.0	99.8
Kipp et al. (15)	2008 USA	Tao Brush/LBC	Hysterectomy	Pre/post	137	83	17	33	4	83.0	89.2
Yanoh et al. (16)	2012 Japan	Uterobrush/ endocyte/ endosearch/ softcyto/tube /cottonswab/NA	Biopsy/D&C	NA	1045	328	25	605	87	92.9	87.4
Remondi et al. (17)	2013 Italy	Endoflower/ LBC	Hysteroscopy and biopsy	Post	98	11	4	82	1	73.3	98.8
Yang et al. (18)	2017 China	SAP-1 sampler/ LBC	D&C	Pre/post	1672	154	167	1286	65	48.0	95.2

*LBC, Liquid - based cytology; NA, not available; D&C, dilatation and curettage*.

### Study Quality

We assessed the quality of eligible studies by QUADAS-2 and found that the quality of all the studies was good (Table 2).

**Table 2:** Risk of bias and concerns of applicability by study using a modified Quadas-2 tool.

	**Risk of bias**				**Applicability concerns**		
	**Patient selection**	**Index test**	**Reference standard**	**Flow and timing**	**Patient selection**	**Index test**	**Reference standard**
Maksem et al. (10)	Unclear	Low	Low	Low	Low	Low	Low
Garcia et al. (11)	Low	Low	Low	Unclear	Low	Low	Low
Papaefthimiou et al. (12)	Low	Low	Low	Low	Low	Low	Low
Andrijono et al. (13)	Unclear	Low	Low	Low	Low	Low	Low
Buccoliero et al. (14)	Unclear	Low	Low	Unclear	Low	Low	Low
Kipp et al. (15)	Unclear	Low	Low	Unclear	Low	Low	Low
Yanoh et al. (16)	Low	Low	Low	Unclear	Low	Low	Low
Remondi et al. (17)	Low	Low	Low	Unclear	Low	Low	Low
Yang et al. (18)	Low	Low	Low	Unclear	Low	Low	Low

### Diagnostic Accuracy

**Figure 3:**
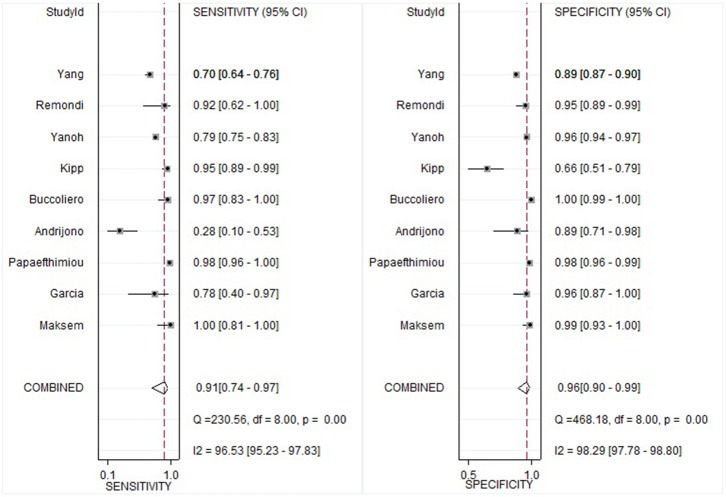
Forest plots of sensitivity and specificity.

The pooled sensitivity and specificity of the cytological method in detecting endometrial atypical hyperplasia or cancer was 0.91 (95% CI 0.74–0.97) and 0.96 (95% CI 0.90–0.99), respectively (Figure 3). The pooled PLR and NLR were 25.4 (95% CI 8.1–80.1) and 0.10 (95% CI 0.00–0.30), respectively. The DOR which used to evaluate the diagnostic test performance,reached 260 (95% CI 36–1905).

### Heterogeneity and Sensitive Analysis

*I*^2^ values of pooled sensitivity and specificity were 96.53 (95% CI, 95.23–97.83) and 98.29 (95% CI, 97.78–98.80), which indicated a statistically significant heterogeneity. A sensitivity analysis was performed to assess the influence of each study, in which each individual study was removed each time. No significant change or reversal of result was found (Table 3). The *I*^2^-value showed that none of the single study affected the heterogeneity of this meta-analysis.

**Table 3:** Sub-analysis and sensitivity analysis on the diagnostic accuracy of endometrial cytological sampling.

**Variables**	**Study number**	**SEN (95%CI)**	**I^**2**^**	**SPE (95%CI)**	**I^**2**^**	**PLR (95%CI)**	**NLR (95%CI)**	**DOR (95%CI)**
**SUBGROUP ANALYSES**								
**Sample size**								
<300	5	0.89 (0.55, 0.98)	96.38	0.93 (0.81, 0.98)	92.26	13.5 (4.2, 43.6)	0.11 (0.02, 0.65)	119 (10, 1359)
≥300	4	0.93 (0.71, 0.99)	99.04	0.98 (0.91, 1.00)	99.62	50.5 (9.0, 284.1)	0.07 (0.01, 0.03)	727 (28, 18689)
**Country**								
Europe	4	0.96 (0.86, 0.99)	76.62	0.99 (0.94, 1.00)	80.56	73.6 (15.4, 351.9)	0.04 (0.01, 0.15)	1769 (160, 19516)
Other	5	0.84 (0.50, 0.96)	93.05	0.92 (0.79, 0.97)	96.41	10.2 (3.6, 29.3)	0.18 (0.04, 0.72)	58 (7, 477)
**SENSITIVITY ANALYSES**								
Maksem et al. (10)		0.88 (0.70, 0.96)	96.03	0.96 (0.87, 0.99)	98.06	21.4 (6.4, 72.0)	0.12 (0.04, 0.36)	172 (24, 1247)
Garcia et al. (11)		0.92 (0.73, 0.98)	97.20	0.96 (0.88, 0.99)	98.61	26.1 (7.1, 95.1)	0.09 (0.02, 0.31)	299 (31, 2864)
Papaefthimiou et al. (12)		0.87 (0.68, 0.96)	95.30	0.96 (087, 0.99)	98.04	22.7 (6.1, 83.9)	0.13 (0.04, 0.38)	174 (22, 1381)
Andrijono et al. (13)		0.93 (0.83, 0.97)	97.19	0.97 (0.90, 0.99)	98.92	30.8 (8.8, 107.7)	0.07 (0.03, 0.19)	435 (65, 2930)
Buccoliero et al. (14)		0.89 (0.69, 0.97)	95.05	0.94 (0.88, 0.97)	97.15	15.7 (6.8, 36.7)	0.12 (0,04, 0.37)	135 (23, 782)
Kipp et al. (15)		0.90 (0.70, 0.97)	96.89	0.97 (0.93, 0.99)	98.41	31.6 (10.8, 92.7)	0.10 (0.03, 0.35)	318 (33, 3031)
Yanoh et al. (16)		0.92 (0.74, 0.98)	97.21	0.97 (0.88, 0.99)	98.46	26.6 (7.1, 99.8)	0.08 (0.02, 0.31)	320 (32, 3158)
Remondi et al. (17)		0.90 (0.71, 0.97)	97.08	0.97 (0.88, 0.99)	98.58	26.6 (7.1, 100.2)	0.10 (0.03, 0.34)	267 (28, 2543)
Yang et al. (18)		0.92 (0.76, 0.98)	96.04	0.97 (0.90, 0.99)	96.69	31.2 (8.7, 111.4)	0.08 (0.02, 0.28)	389 (45, 3397)
Total		0.91 (0.74, 0.97)	96.53	0.96 (0.90, 0.99)	98.29	25.4 (8.1, 80.1)	0.10 (0.03, 0.30)	260 (36, 1905)

*SEN, sensitivity; SPE, specificity; PLR, positive likelihood ratio; NLR, negative likelihood ratio; DOR, diagnostic odds ratio; CI, confidence interval*.

### Subgroup Analysis

Subgroup 1: the corresponding values of the subgroup with sample size <300 were 0.89 (95% CI: 0.55, 0.98) for sensitivity and 0.93 (95% CI: 0.81, 0.98) for specificity. While in subgroup sample size ≥300, the sensitivity was 0.93 (95% CI: 0.71, 0.99) and specificity was 0.98 (95% CI: 0.91, 1.00). Subgroup 2: the corresponding values of the studies of European countries showed the sensitivity of 0.96 (95% CI: 0.86, 0.99) and specificity of 0.99 (95% CI: 0.94, 1.00) and in other countries were 0.84(95% CI: 0.50, 0.96) and 0.92 (95% CI: 0.79, 0.97), respectively (Table 3).

### Publication Bias Analysis

**Figure 4:**
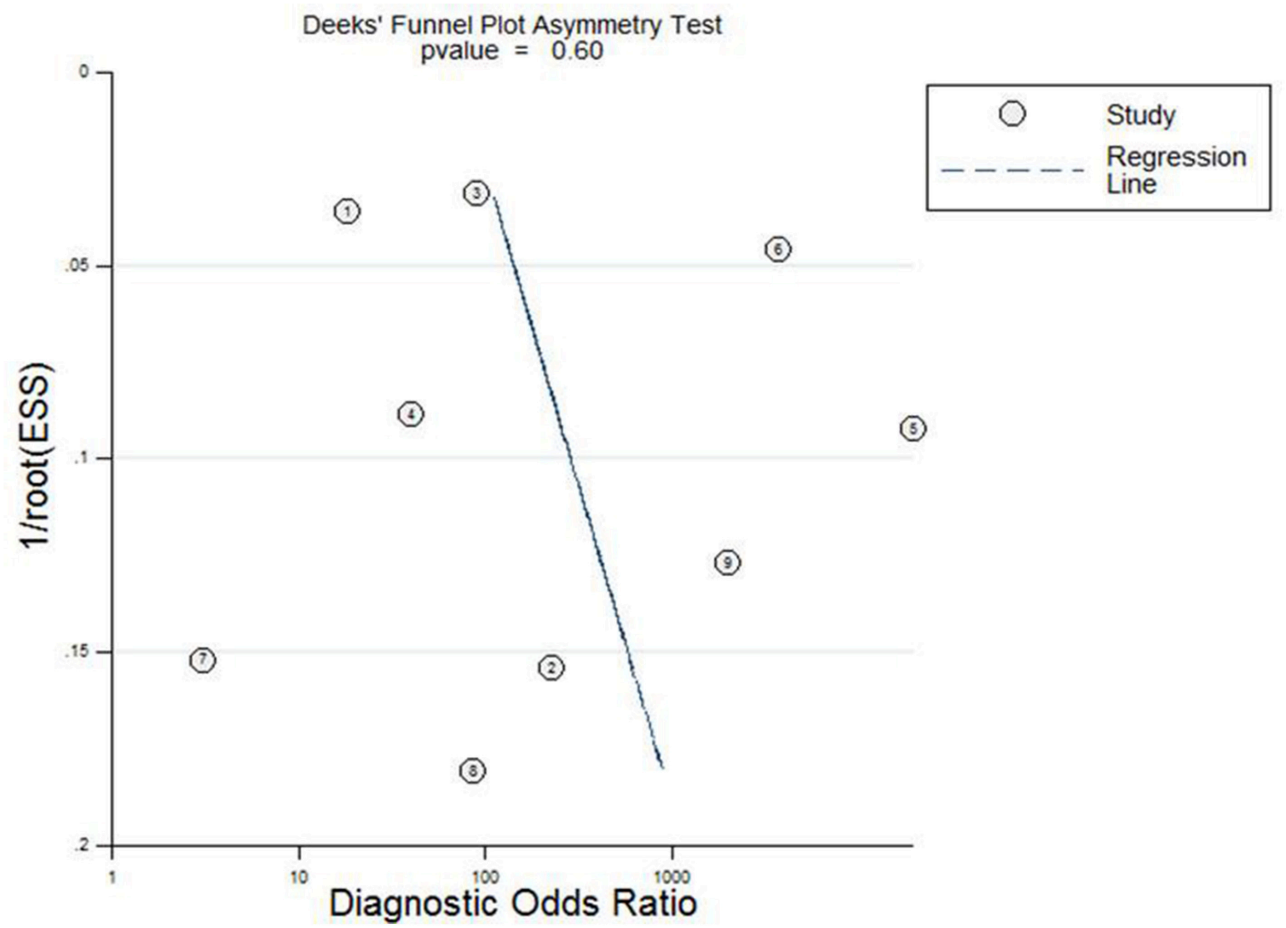
Deeks regression line showed no significant publication bias of studies.

Deeks funnel plot asymmetry test was conducted to evaluate publication bias in this study (Figure 4), which showed statistically nonsignificant publication bias (*P* = 0.60).

### Clinical Utility

**Figure 5:**
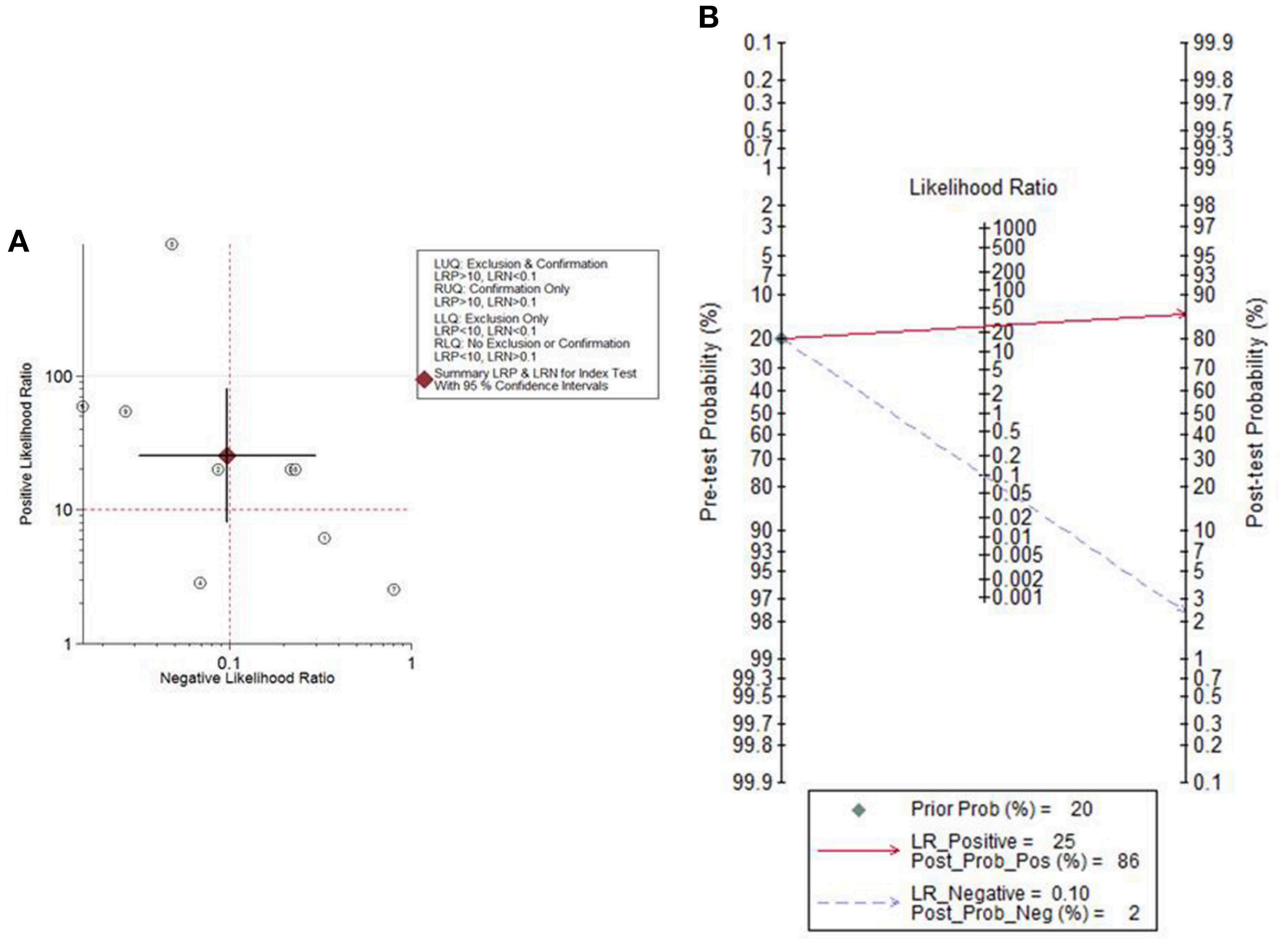
The likelihood ratio matrix and Fagan's plot. **(A)** The likelihood ratio matrix of the cytological method for the detection of endometrial atypical hyperplasia or cancer. **(B)** Fagan's plot presented the clinical utility of the cytological method for the detection of endometrial atypical hyperplasia or cancer.

Given the PLR and NLR, the cytological detection method of endometrial atypical hyperplasia or cancer was located in the left upper quadrant (Figure 5A), indicating that the cytological detection method could serve as a test to confirm and exclude endometrial atypical hyperplasia or cancer. Fagan's plot indicated a dramatic improvement in posttest probability. When the pretest probability of endometrial atypical hyperplasia or cancer was set to 20%, using the cytological method as a source to detect the above diseases could significantly raise the posttest probability of a positive result to 86% and lower the posttest probability of a negative result to 2% (Figure 5B).

## Discussion

It is currently estimated, that 60 million cervical cytology examinations are performed every year in the United States (19). Cytopathological screening, histopathological diagnosis and even the human papillomavirus vaccine are used to prevent and to make early diagnosis of cervical cancer, which helps the early detection and lowers the mortality of cervical cancer. In the absence of such effective screening programs and prevention methods, endometrial malignant diseases are becoming the most prevalent cancer of the female genital tract in developed countries, accounting for nearly 50% of all new diagnoses of gynecological cancer (20, 21). Nearly 75–80% of all endometrial cancer patients diagnosed at early stage (22, 23). But researchers are still paying attention to the early diagnosis of endometrial malignant diseases, especially its precancerous lesion.

Histological (D&C and Pipelle) and cytological diagnosis are two classes of endometrial sampling modalities. Both D&C and the most golden standard of evaluating the endometrium, hysteroscopic-guided uterine biopsy, are painful, expensive, and requires dilatation and anesthesia (24–26). Insufficient samples carry negative ramifications and increase the difficulty for the pathologist (27). An insufficient rate was reported as 6.4% (810/12,745) of curettage and 6.5% (310/4,777) of endometrial biopsy, and multiple factors contributed to such variation, including patient age, parity, endometrial thickness, sampling device, and provider technique. When stratified by age, the insufficient rate was 2.7% in the group of patients under 40 years old (3,454 cases), 5.8% in the group of 40 to 59 years old (11,838 cases), and 14.6% in the group of 60 years and older (2,230 cases) (28, 29). Sakhdari et al. (27) also showed that 15% (226/1,768) of the samples of women age 60 and older were reported as insufficient, and Barut et al. reported the insufficient rate was likely associated with menopause, with 6.5% (26/401) in premenopausal and 49.2% (120/244) in postmenopausal women (25). However, 75% of endometrial cancers occurred in women older than 55 years of age, with a median age of 62 (30). A meta-analysis evaluated the diagnostic rate of D&C and hysteroscopy in postmenopausal women. It pointed out that D&C had a high rate of non-diagnostic samples 31% (range 7–76%) and a high failure rate of 11% (range 1–53%), which lead to a missing diagnosis rate of 7% (range 0–18%) (31).

Pipelle, as another widely used endometrial biopsy apparatus, is safe, cost-effective, and easily preformed (24). A meta-analysis of 39 studies, including 7,914 patients, revealed the concordance rate between Pipelle and D&C/hysteroscopy/hysterectomy in endometrial cancer detection of postmenopausal and premenopausal women was 99.6% and 91%, respectively (32). However, the Pipelle is random point sampling and said to sample 4.2% of the uterine cavity (33), and 25–36% women using Pipelle were found to have insufficient tissue for pathologic assessment (34).

Both D&C and Pipelle have their limitations in detecting endometrial cancer. Hysteroscopic guided biopsy showed a high diagnostic accuracy for endometrial cancer diagnosis (estimated sensitivity of 82.6% and specificity of 99.7%), data from a meta-analysis over 9,000 patients (35), but it could not be performed on asymptomatic women or used as a screening method. Are histological procedures (curettage or biopsy) enough to be the only methods in the diagnosis of endometrial diseases?

Endometrial cytology examination may be an inevitable method for endometrial cancer screening and a combined diagnostic procedure. It might have been hampered by the frequent presence of excess blood, mucus and overlapping cells and varied endometrium cell morphology with different sex hormone levels. However, liquid-based preparation techniques improve the diagnostic accuracy of endometrial cytology (6, 36), and more and more scholars have made efforts on the endometrial cytology reporting system (37–39). With the establishment and maturation of universal standards for the reporting system, endometrial cytology will truly play an important role in the diagnosis of endometrial diseases and endometrial cancer screening. Kondo et al. tested some different methods in 114 consecutive symptomatic women, and they reported that the sensitivity of detecting malignancy increased from 92% to 98% when endometrial cytology was combined with suction curettage (40).

Our meta-analysis showed that endometrial cytology had a high diagnostic accuracy and could serve as a test to confirm or exclude endometrial atypical hyperplasia or cancer. The pooled sensitivity and specificity of the cytological method in detecting endometrial atypical hyperplasia or cancer was 0.91 (95% CI 0.74–0.97) and 0.96 (95% CI 0.90–0.99), respectively. Its diagnostic odds ratio reached 260 (95% CI 36–1905). The pooled positive likelihood ratio and negative likelihood ratio was 25.4 and 0.10, respectively. Therefore, we can conclude that the test results of endometrial cytology are very accurate in diagnosing endometrial atypical hyperplasia or cancer.

Therefore, we recommend that D&C and Pipelle are still practical procedures to evaluate the endometrium, cytological examinations should be utilized as an additional endometrial assessment method, especially for women at high-risk for endometrial cancer.

Additionally, endometrial cytology is inexpensive, tolerated well and can be performed without anesthesia in an outpatient clinic. It is now the most common test for an initial evaluation of endometrial cancer in Japan (7) and has been encouraged as the first level screening method for women at high risk for endometrial cancer (37). Japanese epidemiological data revealed that the overall death rate of endometrial cancer decreased from 20.0 per 100,000 in 1950 to 8.0 per 100,000 in 1999, and this was thought to be a consequence of cytological screening (41).

Many researchers reported a high risk of endometrial cancer with positive cervical cytology (42, 43). Abnormal cervical cytology was associated with high-grade endometrial cancer, worse 5-year median recurrence-free survival and worse disease-specific survival (44). Positive cervical cytology should also be considered as a high risk of endometrial cancer, and endometrial cytology may benefit this kind of patients even with no clinical symptom.

An important strength of this meta-analysis is that we performed a thorough search for articles on the diagnostic accuracy in women with endometrial atypical hyperplasia or cancer using endometrial cytology. This article has several limitations. First, the risk of missing potentially relevant articles is a concern. Otherwise, the relatively small number of studies and variability in methods did not allow for more standard statistical analyses. Higher sensitivity and specificity could be found in subgroup of studies with sample size ≥ 300 and studies in European countries. However, after the subgroup analyses and sensitivity analysis, no factor showed associated with high heterogeneity. Patient age, menopause or not, different kinds of clinical symptoms, varies of cytological samplers and histological sampling methods might contribute to the high heterogeneity, and further study should approve it with enough data. What's more, the studies that are included in the meta-analysis are performed in symptomatic women. More data are needed before endometrial cytology being an effective screening tool for asymptomatic women with high-risks of endometrial cancer.

## Conclusion

In conclusion, endometrial cytology is an efficient diagnostic method and could be applied in the diagnosis of endometrial disorders. The diagnostic accuracy of endometrial carcinoma will surely be improved by the combination of cyto-histopathological procedures and vaginal ultrasonography. Moreover, cytological examination, as a proper outpatient procedure, should be advised for endometrial screening, especial for those with high-risks of endometrial cancer.

## Author Contributions

QingW and QiW drafted the manuscript. QingW, LZ, and CS collected the case. QiW, LH, and SM performed the meta analysis. HH performed the pathological figure. QS helped to revise the manuscript. QL conceptualized the study.

### Conflict of Interest Statement

The authors declare that the research was conducted in the absence of any commercial or financial relationships that could be construed as a potential conflict of interest.
